# Foveolar‐Type Gastric Adenoma With a Raspberry‐Like Appearance That Underwent Dramatic Morphological Changes Within Eight Months

**DOI:** 10.1155/crgm/1594833

**Published:** 2026-05-24

**Authors:** Kohei Funasaka, Tadahiro Yokoyama, Noriyuki Horiguchi, Takahiro Marukawa, Hyuga Yamada, Ryoji Miyahara, Madoka Isomura, Takao Sakai, Sachiko Minamiguchi, Yoshiki Hirooka

**Affiliations:** ^1^ Department of Gastroenterology and Hepatology, Fujita Health University, Toyoake, Aichi, Japan, fujita-hu.ac.jp; ^2^ Department of Diagnostic Pathology, School of Medicine, Fujita Health University, Toyoake, Aichi, Japan, fujita-hu.ac.jp

**Keywords:** case report, endoscopic mucosal resection, FGA-RA, foveolar-type gastric adenoma with a raspberry-like appearance, raspberry-like lesion

## Abstract

**Background:**

Foveolar‐type gastric adenomas with a raspberry‐like appearance in the stomach have recently been identified. However, their etiology and mechanisms of development and growth remain unknown.

**Case Presentation:**

We observed a foveolar‐type gastric adenoma with a raspberry‐like appearance that underwent rapid morphological changes. The first esophagogastroduodenoscopy revealed multiple reddish polyps on the cardia and fundus of the stomach, without *Helicobacter pylori* infection. The second‐largest polyp on the fundus had a red glove appearance and measured 5 mm in diameter. We diagnosed all of them as hyperplastic polyps on the basis of their shape. Given that the patient had received 10 mg of vonoprazan for a long time to treat reflux esophagitis, 20 mg of famotidine was prescribed instead. Eight months later, the largest polyp on the cardia had disappeared.

However, a reddish raspberry‐like lesion was observed at the site of a previously identified polyp on the fundus and was diagnosed as foveolar‐type gastric adenoma by forceps biopsy. The pathological diagnosis was foveolar‐type gastric adenoma with high‐grade dysplasia as determined by tissue resection with endoscopic mucosal resection (EMR).

**Conclusion:**

This is a valuable case report of a lesion evolving from a hyperplastic‐like polyp morphology to a raspberry‐like lesion within eight months.

## 1. Introduction

In 2019, Shibagaki et al. reported 20 cases of reddish foveolar‐type gastric adenomas, which resemble hyperplastic polyps, in the stomachs of patients without *Helicobacter pylori (Hp)* infection [1]. Their appearance is quite different from that of conventional foveolar‐type gastric adenomas, which are whitish flat lesions. Thus, they have uniquely been called “foveolar‐type gastric adenomas with a raspberry‐like appearance (FGA‐RAs).” While most lesions are noninvasive and are predominantly classified as low‐grade dysplasia, approximately 40% of reported cases have been classified as high‐grade dysplasia, which corresponds to noninvasive carcinoma in Japan [2]. Thus, FGA‐RAs are treated with endoscopic mucosal resection (EMR). However, the etiology of raspberry‐like lesions and their mechanisms of development remain unknown. Here, we report a case in which a FGA‐RA developed from a hyperplastic‐like polyp within only eight months.

## 2. Case Presentation

A 60‐year‐old male was referred to our hospital because a large polyp was found on the gastric cardia by esophagogastroduodenoscopy (EGD) during a routine medical checkup. He had a four‐year history of gastric polyps. He had taken 10 mg of vonoprazan once a day to treat his reflux esophagitis. He had variant angina and several metabolic diseases, namely, hyperlipidemia, diabetes, and hypertension. His family history revealed that his father had prostate cancer and his mother had uterine cancer. His alcohol consumption was 1000 mL of beer per day. He stopped smoking five years ago. Blood tests revealed no abnormalities, specifically, negative results for anti‐*Hp* IgG antibodies (< 3 U/mL). There were no remarkable physical findings.

**FIGURE 1:**
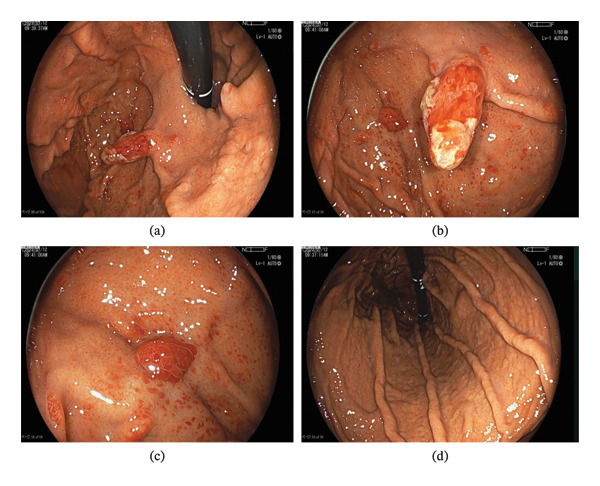
Endoscopic images from the first examination in our hospital. (a) There were several polyps in the cardia and fundus. (b) A 15‐mm reddish polyp on the anterior wall of the cardia. (c) A 5‐mm red glove‐like Y‐III polyp in the fundus. (d) There was no atrophic change in the gastric mucosa.

EGD at our hospital revealed a 15‐mm reddish Yamada type III (Y‐III) polyp with mucus adhesion on the anterior wall in the gastric cardia (Figures 1(a) and 1(b)), a 5‐mm red glove‐like Y‐III polyp on the fundus (Figure 1(c)), and several additional 2‐mm Y‐II reddish polyps. There was no atrophic change in the gastric mucosa (Figure 1(d)). Owing to the typical endoscopic findings of the polyps, we diagnosed them morphologically as hyperplastic polyps without biopsy. We thought that long‐term use of vonoprazan had affected the proliferation of some polyps; thus, we switched the antacid agent to famotidine.

**FIGURE 2:**
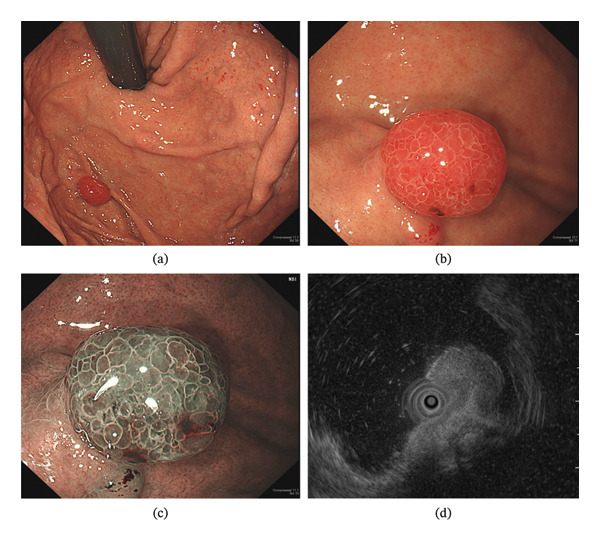
Endoscopic images from the 8‐month follow‐up examination. (a) The 15‐mm red polyp on the anterior wall had disappeared. (b) An 8‐mm raspberry‐like lesion was found where the Y‐III polyp had been previously located on the fundus. (c) Narrow band imaging. (d) Endoscopic ultrasonography.

Eight months later, the patient underwent EGD again to follow up on these polyps. As expected, the hyperplastic polyp found in the anterior wall of the gastric cardia had disappeared with a scar, and the small hyperplastic polyps found in the fundus had also disappeared (Figure 2(a)). However, an 8‐mm raspberry‐like lesion was found where the Y‐III polyp had been previously located on the fundus (Figure 2(b)). Its surface had a heterogeneous papillary structure with entirely different characteristics, and a biopsy revealed foveolar‐type adenoma. Narrow band imaging with magnification revealed heterogeneous papillary structures, and the white zone reflecting the crypt marginal epithelium was clear and thin (Figure 2(c)). Endoscopic ultrasonography revealed that the third layer was partially drawn into the polyp, but no thinning or discontinuity of the third layer was observed (Figure 2(d)). Therefore, we removed this tumor completely by EMR.

**FIGURE 3:**
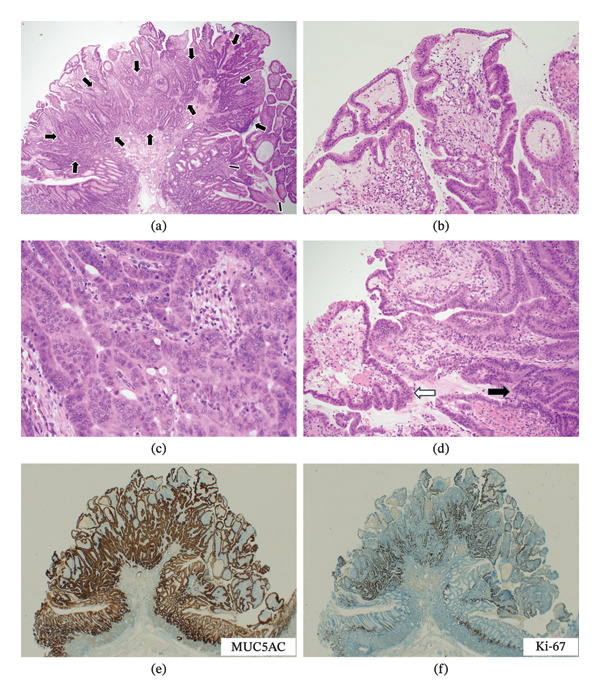
Histopathological findings of foveolar‐type adenoma. (a) Low‐power magnification of the entire polyp. The superficial area shows low‐grade foveolar dysplasia, and deeper regions show high‐grade foveolar dysplasia (between black arrows) (HE, × 20). (b) Superficial areas of low‐grade foveolar dysplasia (HE, × 100). Slightly elongated nuclei with mild atypia are observed. An apical mucin cap is present. Increased and dilated capillaries are observed in the stroma. (c) High‐grade foveolar dysplasia (HE, x200). Glandular disarrangement with irregular branching and fusions of tubules, with nuclear enlargement and stratification, and more frequent mitotic figures. (d) The boundary between high‐grade and low‐grade foveolar dysplasia (HE, × 100). It was difficult to clearly identify the boundary as the atypia appeared to gradually change (black arrow: area of high‐grade dysplasia; white arrow: area of low‐grade dysplasia). (e) MUC5AC. (f) Ki‐67.

A semipedunculated Y‐III polyp was present in the gastric fundic gland region (Figure 3(a)). The lesion was intramucosal with a predominantly papillary architecture. The boundary with the nonneoplastic epithelium was distinct because of nuclear enlargement and papillary features. Superficially, the lesion showed predominantly low‐grade foveolar dysplasia with mildly elongated nuclei and preserved polarity (Figures 3(a) and 3(b)). The apical mucin cap was variably present (Figure 3(b)). The stroma contained increased, dilated capillaries (Figure 3(b)). Deeper portions showed high‐grade foveolar dysplasia with glandular disarray, irregular branching/fusion of tubules, nuclear enlargement and stratification, and more frequent mitoses (Figure 3(c)). The transition between high‐ and low‐grade foveolar dysplasia was clear in some areas but gradual in most of the lesion (Figure 3(d)). Immunohistochemistry revealed diffuse MUC5AC positivity in neoplastic cells (Figure 3(e)), focal MUC6 positivity, and MUC1 and MUC2 negativity. p53 expression showed a wild‐type pattern in both grades. The Ki‐67 labeling index was greater in the high‐grade component (Figure 3(f)). The muscularis mucosa appeared partially indistinct because of the complicated appearance associated with inflammatory changes on hematoxylin‒eosin sections; however, immunohistochemical staining with desmin confirmed that the muscularis mucosa was preserved. No vascular or lymphatic invasion was detected.

The final pathological diagnosis was a 10 mm × 9 mm foveolar‐type gastric adenoma with high‐grade dysplasia (ly0, v0, HM0, and VM0). The patient was discharged without any complications after five days, and there was no evidence of recurrence 15 months after EMR.

## 3. Discussion

In 2019, Shibagaki et al. reported 20 cases of foveolar‐type gastric adenoma without *Hp* infection and with lesions that were bright red and had the characteristic morphology of Y‐III lesions; these cases were called FGA‐RA [1]. Immunohistochemistry staining revealed that FGA‐RAs highly express MUC5AC. The average size of FGA‐RA is small, less than 5 mm, and rarely changes during follow‐up [3]. Since invasive features or metastasis have never been reported [2–4], the clinical course of this neoplasm is likely to be good. In 2024, a single‐nucleotide variant in the KLF4 gene was discovered in raspberry‐type foveolar adenomas [5].

Before Shibagaki suggested the concept of FGA‐RA, several case reports were published describing reddish, elevated lesions located in the upper stomach that coexisted with hyperplastic polyps in the gastric mucosae of *Hp*‐negative patients [6, 7]. The endoscopic images were so similar that we considered them to be raspberry‐like gastric adenomas retrospectively.

To the best of our knowledge, this morphological change from a reddish polyp to a raspberry‐like lesion has never been previously reported.

**FIGURE 4:**
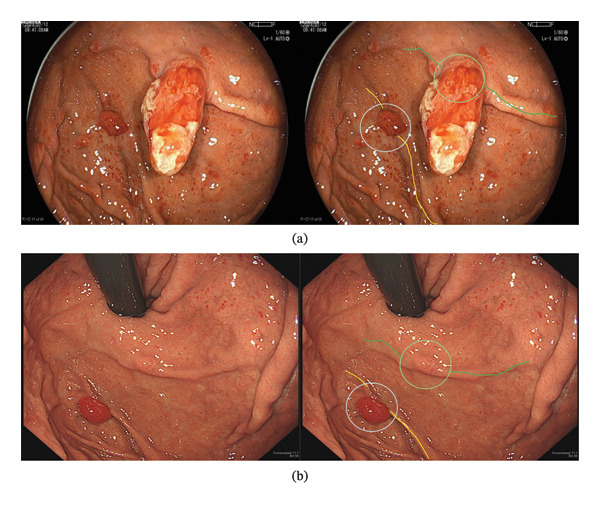
Comparison of endoscopic images between the past polyp (a) and current polyp (b). In the right image, the corresponding folds and polyps are marked with lines (yellow and green) and circles (light blue and yellow green) of the same color.

In our present case, a detailed comparison of past endoscopic images based on the gastric folds revealed that an 8‐mm reddish, glove‐like polyp had transformed into a raspberry‐like lesion (Figures 4(a) and 4(b)). On the other hand, the 15‐mm hyperplastic polyp in the cardia disappeared after cessation of vonoprazan use, and other small polyps and punctate redness of the mucosa also disappeared. We also considered the possibility that the FGA‐RA developed after the disappearance of the previous polyp. However, low‐grade dysplastic columnar epithelium was present on the surface, and high‐grade dysplasia was present deeper within the lesion. Given the morphological changes that occurred over a short period, these pathological findings support the hypothesis that the enlarged lesion resulted from the growth of a high‐grade dysplasia component in the center. However, identifying the border between high‐grade dysplasia and low‐grade dysplasia using magnifying endoscopy was impossible because it was not at the surface but rather within the lesion. On the basis of our hypothesis, we speculate that the prior polyp we diagnosed as a hyperplastic polyp was not a true hyperplastic polyp pathologically but rather a low‐grade dysplastic columnar epithelium. This speculation is meaningful because the origin of the FGA‐RA is unknown.

On the basis of our case, we speculate that some FGA‐RAs may develop from hyperplastic‐like polyps, as these polyps can grow in the U or M regions of the stomach, even in individuals without *Hp* infection. It is well known that proton pump inhibitors can lead to the development or enlargement of hyperplastic polyps [8]. In a report of a 5‐year follow‐up of oral administration of vonoprazan or lansoprazole, the incidence of hyperplastic polyps and the increase in serum gastrin levels were greater in patients who received vonoprazan [9]. Additionally, one case of FGA‐RA developed after 260 weeks of vonoprazan treatment. As expected, the hyperplastic polyps disappeared after vonoprazan was discontinued in the current case. However, a raspberry‐like lesion remained among multiple polyps. These findings are compatible with our hypothesis that the glove‐like polyp was not a true hyperplastic polyp. We cannot deny the effect of the acid‐secreting environment on tumor formation.

## 4. Conclusion

We report a valuable case of FGA‐RA with high‐grade dysplasia that changed its morphology from a hyperplastic‐like polyp within eight months.

## Author Contributions

All the authors contributed equally to the creation of the manuscript, imaging review, and revisions.

## Funding

No funding was received for this publication.

## Consent

Informed patient consent was obtained for publication of the case details from the patient.

## Conflicts of Interest

The authors declare no conflicts of interest.

## Data Availability

The endoscopic and pathological data used to support the findings of this study are available from the corresponding author upon reasonable request.
